# Regional Variation in the Community Nursing and Support Workforce in England: A Longitudinal Analysis 2010–2021

**DOI:** 10.1155/2024/7513374

**Published:** 2024-06-07

**Authors:** Beth Parkinson, Nicky Cullum, Matt Sutton, Katherine Checkland, Peter Bower, Donna Bramwell, Rachel Meacock

**Affiliations:** ^1^Division of Population Health, Health Services Research and Primary Care, School of Health Sciences, University of Manchester, Manchester, UK; ^2^Division of Nursing, Midwifery and Social Work, School of Health Sciences, University of Manchester, MAHSC, Manchester University NHS Foundation Trust, Manchester, UK

## Abstract

**Introduction:**

Shifting care from hospitals into community-based settings is a major policy goal internationally. Community health services in England currently face the greatest workforce shortages of all sectors, threatening the feasibility of this policy. Moreover, little is known about the extent of variation in community workforce provision regionally and how this relates to determinants of need.

**Aim:**

To analyse regional variation in the community services workforce in England between 2010 and 2021.

**Methods:**

We obtained NHS workforce statistics data on the number of nurses and nursing support staff providing community services at each NHS organisation in England, from March 2010 to November 2021. We aggregated the organisation-level data to both regional and national levels, which enabled us to maintain consistent units of analysis across the decade. To examine longitudinal trends and regional variation in workforce provision, we calculated the number of staff per 100,000 population aged 65+ in each region and each period. We then graphed and summarised the variation and examined the correlations with levels of deprivation and rurality.

**Results:**

There was a twofold variation in community services workforce provision between English regions. In November 2021, the number of staff per 100,000 people aged over 64 ranged from 300 in the South West to 697 in the North West. Most regions experienced a reduction in provision between 2010 and 2021, with a 21.2% reduction nationally. East of England experienced the largest reduction of 39.3%, whilst London experienced a 2.1% increase. In November 2021, regions with more deprived populations had higher workforce provision and regions with a larger proportion of residents living in rural areas had lower workforce provision.

**Conclusions:**

The size of the community services workforce has fallen relative to population needs, contradictory to the policy priority to enhance care in the community. There was substantial regional variation in the size of the workforce, which has persisted throughout the decade. Workforce provision was higher in more deprived areas but lower in rural areas, potentially impacting equitable access in rural areas.

## 1. Introduction

Healthcare workers have a fundamental role in the functioning of health systems, yet it is predicted that there will be a global shortage of 18 million health workers by 2030 [1]. England's National Health Service (NHS) is facing the greatest workforce crisis of its history [2], with workforce shortages across all health and care system staffing groups putting services under significant strain. The biggest shortfall of staff is seen in nursing [3, 4], and recent figures have shown that the number of nurses leaving the NHS is at an all-time high [5].

Within the NHS in England, the adult community nursing workforce plays a crucial role in addressing the population's health needs in their homes and local communities, ideally reducing the need for hospital admission. They contribute to the provision of a diverse range of services, including long-term condition management, reablement, wound care, and palliative care. Community health services currently account for approximately 10% of the NHS budget and deliver 100 million patient contacts each year [6].

In line with the goals of health systems internationally, the NHS Long Term Plan reaffirmed the long-standing policy objective of shifting care from hospitals to community-based settings [7], to be achieved by strengthening the provision and efficiency of community services. Moreover, the Fuller report envisages an important role for community nursing services in delivering more integrated care, working with staff in primary care to deliver a wider range of integrated services outside hospitals [8].

An effective community care workforce is required to deliver these plans. Due to the range of services provided by community health services, the workforce is comprised of a range of healthcare professionals, with 73% of those providing direct patient care being nurses and related nursing support roles [9]. However, community health services have been identified as the area experiencing the greatest shortage of nurses in England [10]. Whilst the number of full-time equivalent (FTE) registered nurses working in adult hospital care has increased by around 24% between 2011 and 2021, the number working in community nursing and health visiting has declined by 7% over the same period [11], with the number of community matrons and district nurses declining by 47% [12]. The challenges facing community nursing are so great that they have led to the development of a forthcoming strategic national community nursing plan aiming to strengthen capacity in the community nursing workforce [13]. This forthcoming plan is, however, much delayed.

Whilst the extent of community nursing workforce shortages has been assessed at a national level [11, 12], little is known about how workforce provision varies across the country. Equitable provision of health care is central to the founding principles of the NHS. In recent years, the NHS has committed to stronger action on health inequalities [7], by addressing unwarranted variation in healthcare provision and the relative disparities in access to services [14]. However, the vast majority of research to date has focused on primary and secondary care, with very little evidence on community health services [15]. Inequalities in the regional distribution of the community services workforce could prevent equitable access to services, impact patient experience and outcomes, and widen health inequalities. Regional variation in the community services workforce will also affect the ability of local systems to respond to the policy requirements to deliver a greater proportion of care in the community [8]. Examining whether the provision of these services varies across the country is a vital first step in understanding the sources of variation in care provision.

Social determinants of health are widely recognised as key drivers of health outcomes, health inequalities, and increased healthcare expenditures [16, 17], with environmental and socioeconomic determinants often shown to be the most influential factors determining health [18, 19]. The NHS aims to distribute the resources available to deliver care across geographical areas in a way that takes into account such differences in the need for care [20]. Therefore, examining whether the regional variation in the community services workforce is associated with known drivers of need will provide important data to facilitate the development of meaningful workforce plans, which take account of local needs. We would expect to see higher workforce numbers in areas of deprivation or rurality if services are provided according to need, for example, because people living in rural areas have a higher reliance on community care due to difficulty accessing other healthcare services [21].

In this study, we aim to analyse the trends over time in the size of the community nurse and nursing support workforce in the English NHS between 2010 and 2021. Second, we aim to examine the extent of regional variation and whether this variation is related to two expected determinants of population need: deprivation and rurality [15, 16]. We focus on community nurses and the related nursing support workforce providing community health services to adults, as these services face increasing demands both from an ageing population with increasingly complex healthcare needs and from the introduction of new care models designed to boost out-of-hospital care [7, 22]. Therefore, this analysis will highlight areas with less adequate staffing and ones which may require specific remedial support. This will also give further insights into the factors affecting the ability of the NHS to meet its commitments to tackle inequalities in access to care and provide insights into potential targets for intervention, such as targeted recruitment and regional schemes to attract new staff [23].

## 2. Materials and Methods

### 2.1. Research Design

To examine the trends in the scale and regional variation in community nursing and nursing support workforce provision, we conducted a descriptive longitudinal study. The units of analysis were the whole of England or the seven NHS Commissioning Regions. The analysis presented in this study is part of a larger study aiming to better understand community services in the English NHS and their role in avoiding hospital admissions [24]. The analysis was designed by all authors in consultation with our patient and public involvement panel and following discussions with stakeholders in NHS England.

### 2.2. Data

#### 2.2.1. Workforce

We obtained data from NHS Digital on the number of FTE nurses and nursing support staff listed as delivering community healthcare services, employed by each NHS organisation in England. The data cover the period from 31 March 2010 to 30 November 2021. Data are available on a quarterly basis for the first seven years of the series (31 March 2010 to 31 March 2017) and monthly thereafter (30 April 2017 to 30 November 2021).

The data are taken from NHS workforce statistics, which are extracted from the NHS human resources and payroll system [25]. It is possible that NHS organisations have opened, closed, or merged over the study period, with staff being transferred between organisations. We aggregated the organisation-level data to both regional and national levels, which enabled us to maintain consistent units of analysis across the decade. Regions were classified as the seven NHS England commissioning regions [26].

The NHS workforce statistics data contain the number of FTE staff employed at all NHS organisations working in the speciality of community services. The data contain two main staff groups: “Nurses and health visitors” and “Nursing support staff,” distinguishing between registered and unregistered nursing staff. Registered nurses are clinically qualified healthcare practitioners on the Nursing and Midwifery Council register who have graduated from an accredited training programme [27]. Registered nurses coordinate, plan, and deliver patient care. Nursing support staff include Healthcare Assistants and Nursing Associates, who assist clinical staff in the care of patients, but who do not have professional registration. Due to this study's focus on community services provision for older adults, we excluded nursery nurses, children's nurses, and health visitors as these roles provide services exclusively for children. We also excluded nurses and nursing support staff working in the specialities of Community Learning Disabilities and Community Mental Health services as they serve different populations.

Independent Healthcare Providers play a significant role in the community health service sector in England, but data from non-NHS providers are sparse. To determine the size of the workforce delivering care outside of the NHS, we obtained data from NHS Digital on the number of nurses and nursing support staff working at Independent Healthcare Providers. This data collection began more recently and is therefore only available for the period of 30 September 2015 to 30 September 2021. Data for staff employed by Independent Healthcare Providers are only collected at six-month intervals throughout the series. Due to the way that the data are collected and the data sharing agreements in place, it is not possible to attribute this workforce to regions, so we presented this at the national level only.

#### 2.2.2. Population Statistics

We obtained midyear population estimates of the usual resident population in an area covered by each Clinical Commissioning Group in England from the Office for National Statistics, for the period 2010 to 2020 [28]. Clinical Commissioning Groups were clinically led statutory NHS bodies responsible for the planning and commissioning of healthcare services for their local area. We interpolated these midyear estimates linearly between years to generate estimates for all quarters or months, to correspond with the workforce data availability. Population figures for 2021 were not yet available at the time of writing, so we used estimates from 2020. Clinical Commissioning Groups were assigned to NHS regions using mappings from the Office of National Statistics [29]. From these, we calculated annual counts of the population aged 65+ residing in each NHS region and nationally. This population was chosen because contact rates with community staff increase significantly following age 65, with this being the primary population served by the adult community workforce we examine [30].

#### 2.2.3. Deprivation and Rurality

For each region, we calculated the proportion of the region's aged 65+ population that lives in the most deprived quintile of lower layer super output areas in England, based on the index of multiple deprivation [31]. England is divided into 32,844 lower layer super output areas (LSOAs), which represent a geographical hierarchy designed for the reporting of small area statistics, with a mean population of 1,500 [32]. We then linked LSOAs to Clinical Commissioning Groups, which were then assigned to NHS regions using mappings from the Office of National Statistics [29]. We also calculated the proportion of a region's aged 65+ population that lives in rural areas using the rural-urban classifications of LSOAs [33]. Urban areas are defined as the connected built-up areas identified by Ordnance Survey mapping that have resident populations above 10,000 people, and rural areas are those with fewer than 10,000 people or are open country side [34].

### 2.3. Data Analysis

To examine how the size of the national community service nursing and nursing support workforce has changed over the decade, we presented the longitudinal trends graphically and using summary statistics. We first examined the longitudinal trend over time in the total FTE NHS adult community nurse and nursing support workforce nationally, from March 2010 to November 2021. To examine whether the numbers of nurses and nursing support staff have kept pace with population growth, we also examined this longitudinal trend per 100,000 population aged 65+.

To examine the scale and regional variation in workforce provision, we then presented longitudinal trends in the total FTE NHS nurse and support workforce per 100,000 population aged 65+ in each of the seven NHS regions. This indicates how regional workforce capacity has changed over time, accounting for the size of the populations served.

We presented the number of adult community nurses and nursing support workforce at Independent Healthcare Providers nationally, in terms of both the total FTE and FTE per 100,000 population aged 65+.

Finally, at the regional level, we examined the relationship between the size of the community nurse and nurse support workforce (adjusted for the size of the region's population aged 65+) and two expected determinants of the population need: the level of deprivation and rurality. Specifically, we estimated Pearson's correlation coefficients between the number of FTE community nurses and nursing support staff per 100,000 population aged 65+ and (i) the proportion of a region's aged 65+ population that live in the most deprived quintile of national LSOAs and (ii) the proportion of a region's aged 65+ population living in a rural area. The size of the FTE workforce observed at our most recent time point, 30 November 2021, was used in these analyses. We also used univariate linear regressions to estimate the association between the size of the community services workforce and (i) the level of deprivation and (ii) the level of rurality. From these regressions, we then calculated the expected level of workforce per aged 65+ population for each region given its level of deprivation or rurality.

## 3. Results

### 3.1. National Trends in Community Nursing and Nursing Support Staff Workforce

**Figure 1:**
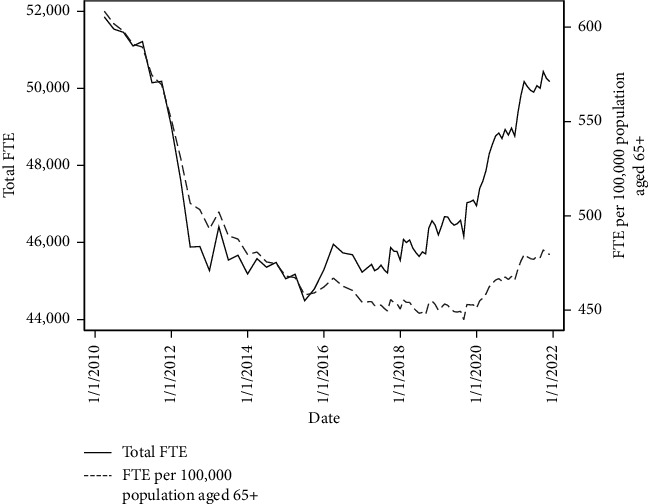
National trends in adult community nurse and nursing support workforce, total FTE, and FTE per 100,000 population aged 65+.

Figure 1 presents the national trends in the number of FTE adult community nurses and nursing support staff employed in NHS organisations from March 2010 to November 2021 (raw numbers and per 100,000 population aged 65+). The size of the workforce decreased substantially during the first half of the decade, with a reduction of 7,400 (14%) total FTE staff between March 2010 and its lowest point in June 2015. When accounting for changes in the size of the older population served, there were 608 FTE staff per 100,000 population aged 65+ in March 2010 (Table 1). This fell by 24.7% to 458 staff per population by June 2015.

Whilst the number of staff started to rise during the second half of the decade, the growth in the size of the workforce since its low point in 2015 did not keep pace with population growth. As of November 2021, there was an average of 480 staff employed per population (Table 1). This figure is 21.2% lower than at the start of the series in March 2010.

In September 2015, Independent Healthcare Providers recorded employing a total of 5,450 FTE nurse and nursing support staff in adult community health service roles, equivalent to 56 FTE per 100,000 population aged 65+ nationally. This means that the size of the workforce reported by Independent Healthcare Providers was approximately 12.2% of the 459 staff per population employed by NHS organisations in September 2015. The number of staff employed by Independent Healthcare Providers had increased by 9% to 61 staff per population nationally in September 2020, which is equivalent to 13.1% of the 465 staff per population employed by NHS providers on that date.

### 3.2. Regional Trends in Community Nursing and Nursing Support Staff Workforce

**Figure 2:**
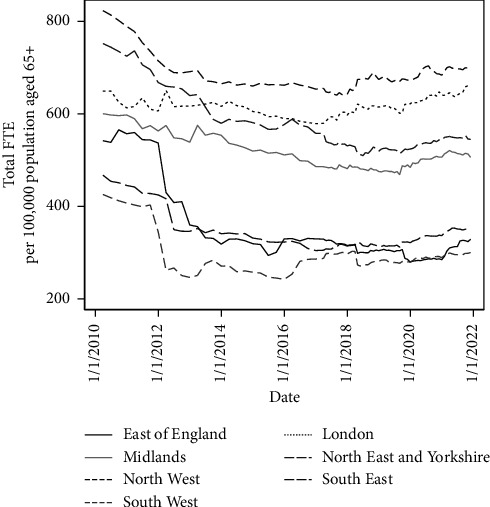
Regional trends in adult community nurse and nursing support workforce, FTE per 100,000 population aged 65+.

There are stark regional differences in the size of the community nursing and nursing support workforce (Figure 2). Every year, there was around a twofold difference between some regions in the number of FTE staff employed per 100,000 population aged 65+. As of November 2021, there were 697 staff per population in the North West compared with just 300 in the South West of England (Table 1).

Regions experienced relatively similar rates of growth in the size of the population aged 65+ (Table 1, column 6), but much more variable changes over time in FTE staff numbers (Table 1, column 3). Regions therefore experienced vastly different trends in workforce per population over the period we examined. Most regions experienced a fall in staff in the first half of the decade to various extents (Figure 2), as seen in the national trends (Figure 1). However, London did not experience this initial drop, with workforce numbers instead staying relatively stable up until 2017 when they began to increase, resulting in a 2.1% increase in workforce per population across the period we examine. Conversely, the East of England experienced the most dramatic drop in workforce numbers at the start of the decade, with workforce numbers then remaining low for the rest of the period. The size of the workforce in the East of England fell from 542 to 329 staff per population between 2010 and 2020, representing a 39.3% reduction in workforce provision. London was the only region where the size of the workforce kept pace with the growth in the size of the older population served. All other regions saw reductions in the size of their workforce per population over the period we examine, ranging from −15.4% in the North West to −39.3% in the East of England.

### 3.3. Regional Workforce and Deprivation

**Figure 3:**
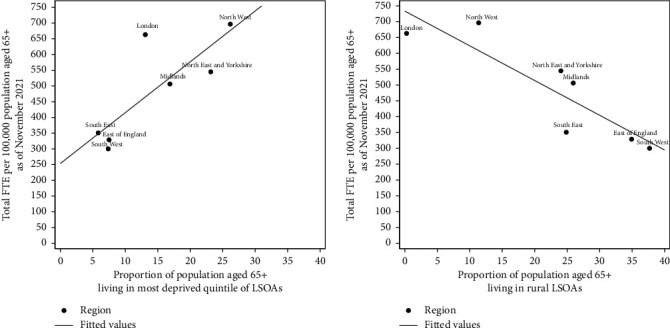
Relationship between adult community nurse and nursing support workforce size and deprivation and rurality.

Deprivation levels vary substantially across the regions of England. In the South East, 5.8% of the population aged 65+ reside in the most deprived quintile of national LSOAs, compared to 26.2% of the aged 65+ population in the North West (Table 1). There was a strong positive correlation between the regional FTE workforce per 100,000 population aged 65+ and the proportion of this older population living in the most deprived LSOAs (Pearson's correlation coefficient = 0.81, *p* value: 0.028), as of November 2021. Regions with a greater proportion of their aged 65+ population living in deprived areas had higher levels of staffing (Panel 1, Figure 3), as demonstrated by the strong positive gradient observed in the line of best fit. Most regions fit this trend relatively closely except London and the North East and Yorkshire. London has 198 more staff per population than expected from the univariate regression, whilst the North East and Yorkshire has 82 fewer staff per older population than expected given the region's deprivation levels.

### 3.4. Regional Workforce and Rurality

The proportion of people residing in rural areas also varies across the regions of England. In the predominantly urban region of London, just 0.3% of the population aged 65+ reside in a rural area (Table 1). The South West has the highest levels of rurality, with 37.7% of the population aged 65+ residing in a rural area. There was a strong negative correlation between the regional FTE workforce per 100,000 population aged 65+ and the proportion of the older population living in rural LSOAs, as of November 2021 (Pearson's correlation coefficient = −0.88, *p* value: 0.008) (Panel 2, Figure 3). The East of England and the South West are the two most rural areas, with over one-third of their older populations living in rural LSOAs. These regions had just under half the workforce per population compared to the two least rural regions, London and the North West.

## 4. Discussion

### 4.1. Summary of Findings

Nurses play a vital role in promoting equitable and essential care, yet workforce shortages and inequitable geographical distributions of staffing pose challenges for health systems across the globe [35]. Most evidence to date has focused on understanding the nursing workforce in acute settings rather than nursing in the community. An effective community nursing workforce is required to support the delivery of the new models of care designed to enhance care provided outside of hospital, yet little is known about trends in the community services workforce within the English NHS and how this varies across the country. Our analysis demonstrates how the community nurse and nursing support staff workforce has changed over time in England, how it varies regionally after accounting for the size of the potential populations served, and how workforce provision is related to two factors expected to reflect higher levels of need amongst the population: deprivation and rurality.

There was a significant decline in the first half of the decade in the number of nurses and nursing support staff providing adult community health services in NHS organisations. Although there was growth in staffing in the later part of the decade, the number of FTE staff was still lower in 2021 than in 2010 after adjusting for the size of the population aged 65+ served. The growth in the size of the workforce since its plateau in mid-2015 has failed to keep pace with population growth, resulting in 21% fewer FTE staff per population at the end of the series in November 2021. In addition to population growth, patient complexity and need are also known to have increased during this period [36], meaning that the demands placed on the workforce have likely grown even further than is reflected by simple population counts.

Some of this initial decline may have been a result of the “Transforming Community Services” programme, which was implemented in March 2011 and required Primary Care Trusts (PCTs) to separate their provider and commissioning responsibilities for community services [37]. In response, the provision of community services was transferred to a range of organisations. The majority was transferred to NHS organisations through the creation of standalone NHS Community Trusts or merged with existing Acute or Mental Health NHS Trusts. The remainder was transferred to organisations outside of the NHS, such as voluntary and independent sector providers [38]. However, the resulting shift of some staff to Independent Healthcare Providers is unlikely to have been large enough to fully explain the decline seen within NHS organisations. Whilst we do not have data on the Independent Healthcare Provider workforce before September 2015, we find that the total size of their community nurse and nursing support workforce recorded is just 12% of the NHS workforce during the period we can observe, at around 5,450 FTE staff in September 2015. The recorded Independent Healthcare Provider workforce was therefore not large enough to account for the observed reduction of 7,400 FTE staff employed by NHS organisations from March 2010 to June 2015.

We find stark variations in the levels of adult community nurses and nursing support workforce in different areas of the country. There was a twofold difference in the number of FTE staff between regions after controlling for the size of the older populations they serve. Regions also experienced different trends in their workforce over the series. Whilst staffing levels per 100,000 population aged 65+ remained relatively consistent across the series in London, this was the only region not to experience a decline in the size of its NHS workforce after adjusting for population growth. FTE adjusted for population size was 2.1% higher in London at the end of the series, whereas the East of England experienced a reduction of −39.3% in the size of its population-adjusted workforce.

Regional variations in the size of the adult community nurse and nursing support workforce are strongly patterned according to the level of deprivation experienced by the regions' populations. Deprivation is a key determinant of community service need [30], so this suggests that regional variations are warranted as they correlate closely with this indicator of need. However, the magnitude of this difference is striking and is contrary to the inverse care law, which has been shown to exist in many other care settings [39]. For example, the provision of general practitioners in an area has previously been found to be inversely related to the deprivation of the population [40]. This suggests that, unlike other healthcare sectors, the community workforce aligns better with deprivation, at least at the regional level. There are also some regions for which this variation is not well explained. London, for example, appears to be an outlier to this trend, having a much higher FTE workforce per population relative to its deprivation levels. This would suggest London may be most able to prioritise the expansion of community services following the recent policy agenda.

Whilst workforce provision follows a positive relationship with levels of deprivation, we find that regional variations are inversely related to rurality. This is in part because deprivation and rurality are themselves moderately negatively correlated. However, if rurality was independently positively associated with workforce provision, we would not expect the regional variation to follow deprivation levels only. A survey of community nurses found that teams serving predominantly rural areas covered an average area of a 17-mile radius compared to 10 miles in urban areas [41]. It has therefore been argued that rural areas require more staffing to account for additional travel distances and the resulting lower proportion of overall time spent with patients associated with serving more remote areas and dispersed populations [42]. Furthermore, people living in rural areas have a higher reliance on community care due to difficulty accessing other healthcare services [21]. However, we observe the opposite relationship when examining workforce provision across England; the two regions with the largest provision of adult community nurses and nursing support workforce per head are those with the lowest proportion of their older population living in rural LSOAs. Our results suggest that populations in rural areas may face inequalities in access to, and provision of, adult community services. This in turn could compound the inequalities in access to other healthcare services known to exist in rural areas, particularly in secondary care, with travel time to the nearest hospital double for people residing in rural compared to urban areas [21].

### 4.2. Comparison with Previous Research

We are not aware of any published analyses of the regional variation in the distribution of the adult community nurse and nursing support workforce. Our findings are, however, in line with existing research that has demonstrated the existence of inequalities in the distribution of healthcare workers internationally. Studies have demonstrated inequalities in the density and distribution of nurses between countries [43, 44] and at a subnational level within 58 countries [45]. However, these studies did not focus specifically on community nursing. Previous studies have also found substantial subnational geographic variation in the distribution of general practitioners internationally [46–48] and within the English NHS [49, 50]. The previous finding that fewer general practitioners are employed in practices in more deprived areas in England [40] contrasts with the findings of our study, where we find the opposite relationship between adult community workforce provision and deprivation. This suggests that the community workforce may align better with deprivation, in contrast with the primary care workforce.

Previous descriptive analysis of NHS workforce statistics shows that the number of nurses and nursing support staff working in acute adult care settings in NHS hospitals increased by 24% nationally, between December 2011 and December 2021 [11], whilst the number working in either community or health visiting decreased by 7% nationally. However, importantly, these analyses combined adult and children's community services, did not account for population growth, and did not examine regional variations as we did in this study.

Recent studies have assessed the trends in the size and composition of the wider primary care workforce in the English NHS. Between 2015 and 2019, there was a 2.9% increase in the total workforce employed at general practices per 1,000 patients, combined with an expanding skill mix of the general practice workforce [51]. The number of practice nurses per 1,000 patients remained fairly constant, but there was an increase in the number of advanced nurse practitioners [51]. However, this study only examined the primary care workforce from 2015 onwards, which is the period from which we observe the size of the community workforce to begin to increase again following a decrease in the first half of the decade.

The number of staff employed at general practices was also found to vary between regions of England [52]. The lowest number of primary care staff per 1,000 patients was seen in the London and South East regions of England [52]. Whilst we do not examine all types of community health service staffing, we do find the South East to be amongst the regions with the lowest community nurse and nursing support workforce. The regions with the highest number of primary care staff were those in the South West and North East [52]. We also show that the North East of England had levels of community nursing and support staff above the national average.

### 4.3. Strengths and Limitations

This is the first national and regional analysis of trends in the number of nurses and nursing support staff employed to provide adult community services in NHS organisations in England. Previous research has focused on either the entire NHS nursing workforce employed across all care settings or only subsets of the community services workforce, such as district nursing, and none have assessed regional variations in the community services workforce. None have examined geographical variation, or how provision relates to deprivation and rurality. We have a long time series containing over 10 years of data, which covers several important policy changes implemented in community health care.

Whilst most community healthcare services are provided by NHS organisations, a proportion is provided by Independent Healthcare Providers. We present data on the number of nurses and nursing support staff providing adult community services recorded by Independent Healthcare Providers nationally. These data are not available for the full period that we have access to data from NHS organisations, starting instead from September 2015. Furthermore, there is an agreement with some Independent Healthcare Providers that data will not be disaggregated below the national level; therefore, we are unable to examine the regional differences in community services provision from Independent Healthcare Providers. It is therefore possible that the regional distribution of the adult community nurse and nursing support workforce employed by Independent Healthcare Providers could explain some of the observed regional variation in the NHS community workforce that we detect. However, the reported size of the workforce employed by Independent Healthcare Providers is not large enough at the national level to explain the reductions in the NHS workforce observed during the period we examine.

Due to how NHS workforce data are collected by NHS Digital, we are unable to capture the wider allied healthcare professional and medical workforce employed in the community setting. However, 73% of clinical staff providing community services are nurses or nursing support staff, so we can capture the majority of the workforce providing care in this setting [9].

## 5. Conclusions

The size of the community nurse and nursing support workforce has fallen relative to population needs, contradictory to the long-standing policy priority of enhancing care in the community. There was substantial regional variation in the size of the workforce, which has persisted throughout the decade. Workforce provision was higher in more deprived areas, but lower in rural areas.

These results focus on the nursing and nursing support workforce as data on alternative roles such as doctors and allied health professionals were unavailable. However, this remains an important avenue for future research and should be the focus of future data collection efforts. Furthermore, the need for consistent units of analysis required us to aggregate the data to the regional level; however, there may be wider inequalities at smaller geographical area levels [51, 52].

### 5.1. Implications for Nursing Management

Boosting out-of-hospital care through increased investment in community health services has been a key policy goal of the NHS for several years. Similarly, the NHS Long Term Plan committed to more action on health inequalities by addressing unwarranted variation in care provision. The findings of this study highlight the need for further action towards addressing community health services nursing workforce shortages nationally and particularly in rural regions, where levels of provision are lower than urban counterparts, potentially preventing equitable access for rural populations.

The failure of size of the workforce to keep up with population growth means that the community nursing workforce will be more stretched and under more pressure than ever before. Therefore, the insights gained from this study in terms of the scale and patterns of inequalities can be used by policymakers to effectively plan nurse recruitment and retention programmes and improve the sustainability and equity of community health services.

## Figures and Tables

**Table 1:** Community nurse and nursing support staff workforce and population size and characteristics.

Region	FTE staff			Population aged 65+			FTE staff per 100,000 population aged 65+			The proportion of the aged 65+ population living in deprived areas in November 2021	The proportion of aged 65+ population living in rural areas in November 2021
	Mar 2010	Nov 2021	Change (%)	Mar 2010	Nov 2021	Change (%)	Mar 2010	Nov 2021	Change (%)		
East of England	5,609.23	4,260.93	−24.04	1,035,031	1,295,979	25.21	541.94	328.78	−39.33	7.50	34.92
London	5,804.98	7,284.70	25.49	893,928	1,098,453	22.88	649.38	663.18	2.13	13.08	0.26
Midlands	9,977.09	10,277.59	3.01	1,661,849	2,030,374	22.18	600.36	506.19	−15.69	16.87	25.94
North East and Yorkshire	10,742.15	9,160.58	−14.72	1,428,503	1,681,146	17.69	751.99	544.90	−27.54	23.16	24.03
North West	8,724.48	9,167.87	5.08	1,059,811	1,316,247	24.20	823.21	696.52	−15.39	26.17	11.37
South East	6,659.30	6,210.24	−6.74	1,424,852	1,770,422	24.25	467.37	350.78	−24.95	5.83	24.86
South West	4,334.71	3,815.70	−11.97	1,018,242	1,271,398	24.86	425.70	300.12	−29.50	7.37	37.68
England total	51,851.94	50,177.60	−3.23	8,522,216	10,464,019	22.79	608.43	479.53	−21.19	14.47	23.46

## Data Availability

NHS workforce data are publicly available here, at one level of aggregation higher than we utilise: https://digital.nhs.uk/data-and-information/publications/statistical/nhs-workforce-statistics. We were provided with a longitudinal community services specific subset of these data upon request from NHS Digital. All other datasets are publicly available, and sources are cited throughout the manuscript.
